# microRNA Expression Patterns Reveal Differential Expression of Target Genes with Age

**DOI:** 10.1371/journal.pone.0010724

**Published:** 2010-05-20

**Authors:** Nicole Noren Hooten, Kotb Abdelmohsen, Myriam Gorospe, Ngozi Ejiogu, Alan B. Zonderman, Michele K. Evans

**Affiliations:** 1 Laboratory of Cellular and Molecular Biology, National Institute on Aging, National Institutes of Health, Baltimore, Maryland, United States of America; 2 Clinical Research Branch, National Institute on Aging, National Institutes of Health, Baltimore, Maryland, United States of America; 3 Laboratory of Personality and Cognition, National Institute on Aging, National Institutes of Health, Baltimore, Maryland, United States of America; Roswell Park Cancer Institute, United States of America

## Abstract

Recent evidence supports a role for microRNAs (miRNAs) in regulating the life span of model organisms. However, little is known about how these small RNAs contribute to human aging. Here, we profiled the expression of over 800 miRNAs in peripheral blood mononuclear cells from young and old individuals by real-time RT-PCR analysis. This genome-wide assessment of miRNA expression revealed that the majority of miRNAs studied decreased in abundance with age. We identified nine miRNAs (miR-103, miR-107, miR-128, miR-130a, miR-155, miR-24, miR-221, miR-496, miR-1538) that were significantly lower in older individuals. Among them, five have been implicated in cancer pathogenesis. Predicted targets of several of these miRNAs, including PI3 kinase (PI3K), c-Kit and H2AX, were found to be elevated with advancing age, supporting a possible role for them in the aging process. Furthermore, we found that decreasing the levels of miR-221 was sufficient to cause a corresponding increase in the expression of the predicted target, PI3K. Taken together, these findings demonstrate that changes in miRNA expression occur with human aging and suggest that miRNAs and their predicted targets have the potential to be diagnostic indicators of age or age-related diseases.

## Introduction

Human aging is a highly complex process that is characterized by an increase in age-associated diseases such as cancer, type 2 diabetes mellitus, autoimmunity, infections, cerebrovascular and cardiovascular disease. Important in the study of aging is the discovery of new biomarkers that serve as indicators of tissue age and development and that also can aid in the diagnosis of age-related diseases. Studies in model systems suggest that longevity can be modulated by changes in gene and protein expression. In addition, it is widely believed that factors such as calorie restriction may extend the lifespan of organisms, in part, by modulating the levels and expression of particular genes and pathways [1], [2].

Recent evidence suggests that microRNAs (miRNAs) are key regulators of gene expression. miRNAs are small non-coding (∼22 nt) RNAs that incorporate into the miRNA-induced silencing complex (RISC) [3]. This complex typically negatively regulates gene expression through mRNA degradation, translation inhibition or by performing both functions [4], [5]. Accumulating data suggest that miRNAs are important regulators of a variety of cellular processes including cell proliferation, survival, differentiation and replicative sensescence [6], [7].

Extensive research in *Caenorhabditis elegans* has uncovered a role for miRNAs in controlling lifespan [8]. For example, reducing the activity of the *C. elegans* miRNA, lin-4, shortens lifespan. Conversely, increasing the activity of lin-4 lengthens lifespan [9]. Further evidence implicating lin-4 in modulating lifespan includes experiments showing that lin-4 affects lifespan in part through repression of its target lin-14. In addition, lin-14 has an opposite effect on lifespan when compared to lin-4 [9]. More recently, a genome-wide transcriptional profile of miRNAs in *C. elegans* showed that the expression of approximately one third of the miRNAs is modulated during the lifespan [10]. Furthermore, the majority of these age-regulated miRNAs were found to be downregulated in older animals. In addition to *C. elegans*, changes in miRNA expression occur with aging in the mouse liver and brain [7], [11], [12], [13]. Most recently, differences in microRNA abundance were observed in the liver from the Ames dwarf mouse, which have extended longevity compared to wild-type mice [7], [11], [12]. These data suggest that miRNA expression patterns change with the lifespan of model organisms and indicate that different levels of miRNAs may directly affect the aging process.

Recent studies have suggested that changes in miRNA expression also occur with human cellular senescence, an *in vitro* model system that recapitulates certain aspects of aging and cancer [14], [15]. Using two different human cells lines, Brosh and colleagues reported that the onset of cellular senescence, defined as an irreversible decline in cell proliferation after a finite number of divisions in culture, significantly decreases miRNA expression [16]. Furthermore, modulation of two groups of miRNAs has been shown to affect senescence *in vitro.* These include miR-106b, miR-93, miR-25 and miR-15b, miR-24, miR-25, and miR-141 [16], [17]. These subsets of miRNAs and others appear to target different senescence-associated genes, including several that encode cell cycle proteins (the miR-106b group), MKK4 (miR-15b, miR-24, miR-25, miR-141), p16^INK4a^ (miR-24), and IL-6/IL-8 (miR-146a/b) [16], [17], [18], [19]. Importantly, several of these target genes have been shown to be elevated in human tissues from older individuals, suggesting that changes in miRNA expression may modulate key targets to drive senescence.

Research in this area has largely focused on dissecting miRNAs that are important for age-related diseases such as cancer. Little is known about the role of miRNAs in mammalian aging. Here, we have profiled miRNAs expressed in both young and old individuals and identified those that are differentially expressed in older individuals. Furthermore, we report that several putative targets of miRNAs including, PI3K, H2AX and c-Kit are upregulated with age in humans. Thus, changes in the expression of miRNAs and their predicted targets occur with the aging process, and may serve as potential biomarkers of susceptibility to age-associated diseases.

## Results

### Changes in miRNA expression in young versus old individuals

**Figure 1:**
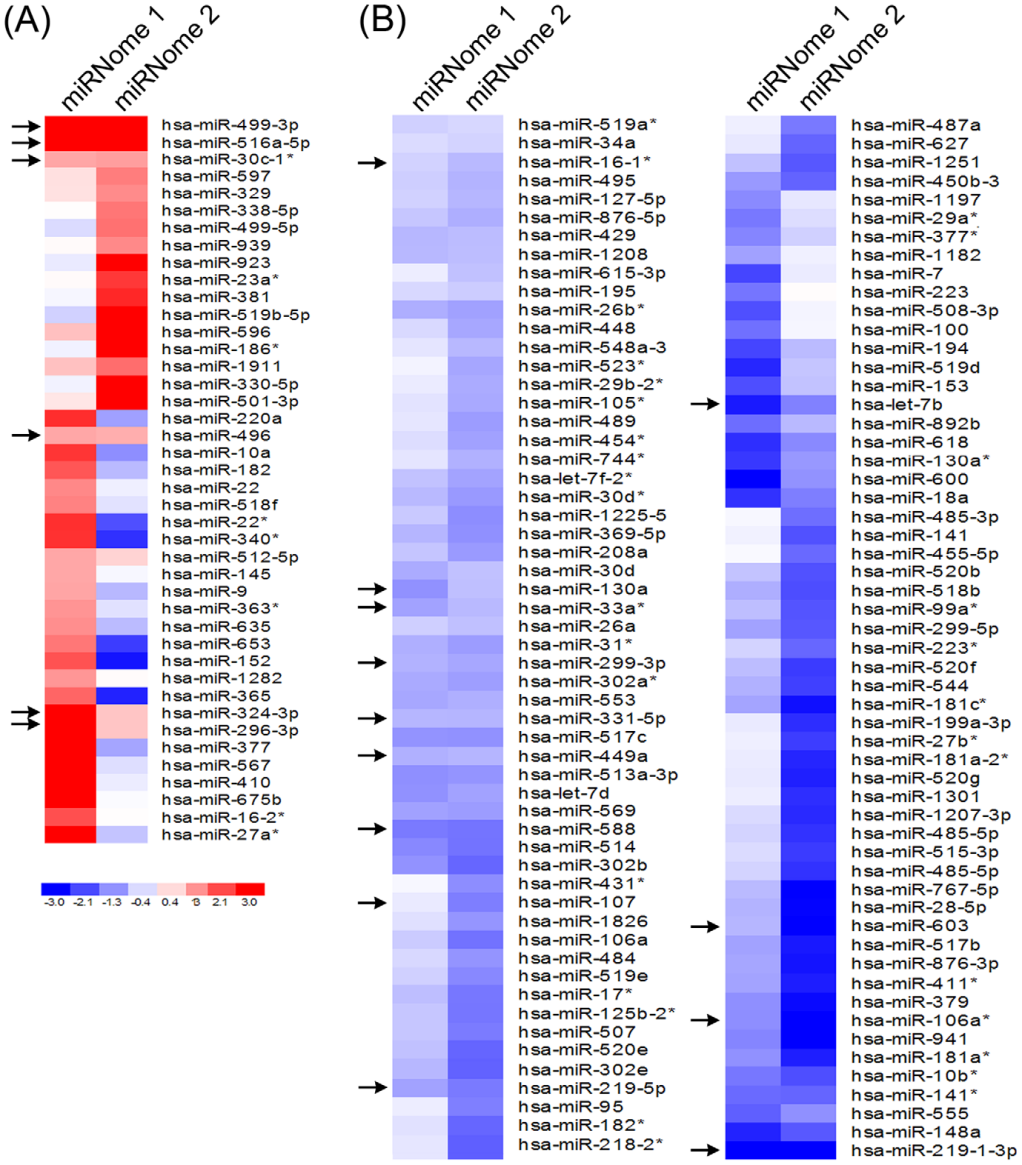
Downregulation of miRNA expression in old individuals. For each panel, one young 30- year-old participant and one old 64-year-old participant were screened for miRNA expression using a real-time RT-PCR based miRNome miRNA profiler kit as described in Materials and Methods. Different pairs of individuals were used for both miRNome 1 and miRNome 2. The heat map was generated using dChip software and indicates the fold log_10_ relative change in expression in young versus old participants[49]. The top upregulated and downregulated miRNAs are shown. Blue indicates downregulation of miRNA expression in the old individuals and red indicates upregulation of miRNA expression in the old individuals. Each patient was sex and race matched as indicated in Table 1. A detailed spreadsheet of the data is available as supplementary information (Table S1). * next to the microRNA refers to the minor form of the miRNA.

To examine whether miRNA expression is altered with age in a human population, we obtained peripheral blood mononuclear cells (PBMCs) from young (30 year old) individuals and old (64 year old) individuals. Demographic data for these subjects are presented in Table 1A. We employed the miRNome miRNA profiler assay, that uses real-time RT-PCR to screen over 800 human miRNAs, in 2 different sets of young and old participants (miRNome 1 and miRNome 2) (Table 1A). Interestingly, we found that most miRNAs are downregulated in older participants compared to younger participants, which is consistent with microarray data from both *C. elegans* and senescent cells *in vitro* (Fig. 1)[10], [16].

**Table 1:** Patient demographic information.

**(A)**	**miRNome**	Young	Old
	n	2	2
	Age	30	64.5
	Race	W	W
	Sex	M	M
**(B)**	**Validation**	Young	Old
	n	14	14
	Age	30.1±0.3	64.2±0.4
	Race	8 W, 6 AA	8 W, 6 AA
	Sex	8 F, 6 M	8 F, 6 M
	Race and Sex	5 W-F, 3 AA-F	5 W-F, 3 AA-F
		3 W-M, 3 AA-M	3 W-M, 3 AA-M

Demographics for individuals in this sub-cohort of the HANDLS study. Age is reported as the mean ± SD. Abbreviations are as follows: W, White; AA, African American; F, Female; M, Male.

**Figure 2:**
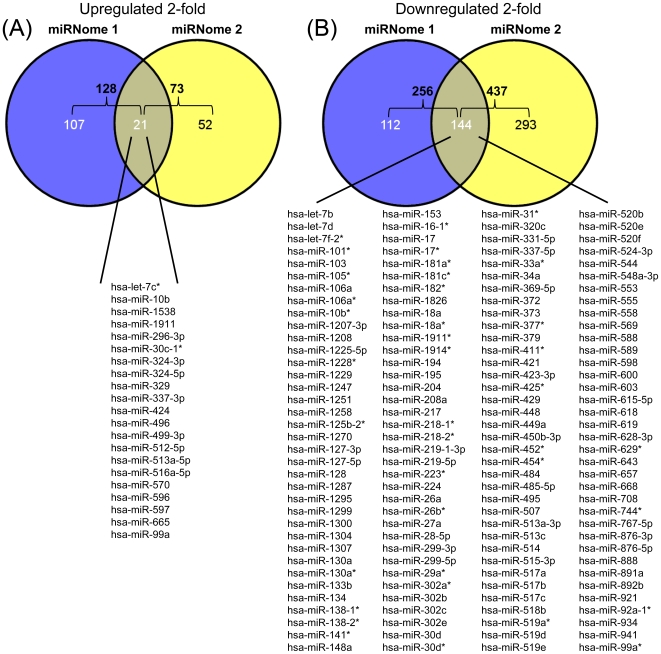
Upregulated and downregulated miRNAs in young and old individuals. A Venn diagram showing miRNAs that expression was either upregulated >2-fold (A) or downregulated >2-fold (B) in the old participants compared to young participants from the two different miRNome analyses in Figure 1. Listed are the miRNAs that overlapped between these 2 different data sets. The complete miRNome analysis for the listed miRNAs is available (Table S2).

We then compared the differentially regulated miRNAs that we found in our two different miRNome experiments. Of the 128 and 73 miRNAs found to be upregulated 2-fold in older patients in miRNome 1 and miRNome 2, 21 overlapped between the data sets (Fig. 2, Table S2). 256 and 437 miRNAs were at least 2-fold lower in miRNome 1 and 2, respectively. Of these miRNAs 144 were found to overlap between datasets, indicating that in PMBCs approximately 16.5% of miRNAs studied were reduced by 2-fold with age (Fig. 2, Table S2).

### Downregulation of miRNAs with human age

**Figure 3:**
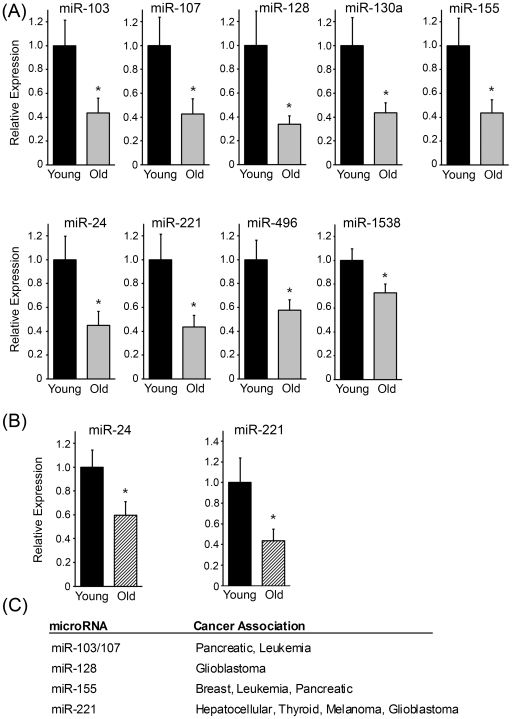
Real-time RT-PCR validation of changes in miRNA expression in young and old participants. (A) Expression of miRNAs from the miRNome analysis were further validated in 14 young and 14 old subject PBMCs (see Table 1B for detailed demographic data) using real-time RT-PCR as described in Materials and Methods. The histograms show normalized averages ± SEM from duplicate experiments. *P<0.05 by both Student's t-test and three-way ANOVA. (B) miR-24 and miR-221 expression was examined in 14 young and 14 old individuals using a TaqMan microarray assay as described in Materials and Methods. (C)Age-associated miRNAs that have been implicated in various cancers. See recent reviews for details [4], [35], [50].

To determine whether the changes in miRNA expression observed with age are consistent in a larger number of individuals, we validated the levels of a number of miRNAs in 14 young and 14 old participants (see Table 1B for demographics). The most upregulated and downregulated miRNAs within our miRNome analysis and other aging-associated miRNAs were chosen for further analysis. Out of the 32 miRNAs tested, 9 were significantly downregulated in older participants (Fig. 3A). The levels of several miRNAs were not significantly changed when we analyzed them in this larger group (Fig. S1). In general, the miRNA expression patterns in the individuals we examined by miRNome analysis showed similar magnitudes of change when we examined them along with the larger cohort. However, two miRNAs (miR-496, miR-1538) were found to be upregulated in the old participants by miRNome analysis and in the validation experiments, but when examined among the larger cohort these miRNAs were found to be downregulated in older participants (Fig. 3A, Table S1, S3). Furthermore, downregulation of miR-24 and miR-221 (two miRNAs we examine further below) was also observed when we measured their expression using a TaqMan microRNA assay (Fig. 3B).

To address whether the changes in miRNA expression may be due to age-dependent differences in subsets of lymphocytes, we examined different T cell markers, which represent a population of lymphocytes that undergo some age-dependent changes [20], [21], [22], [23]. Consistent with published reports for our age groups, we did not find any significant changes in the CD4, CD8, CD4/CD8 ratio or IL-7 receptor expression between young and old participants in our cohort (Fig. S2) [20], [21], [22], [23]. Taken together, these observations and the fact that we observe significant changes in miRNA expression suggest that the changes in miRNA expression are unlikely to be due to the modest effects observed in different lymphocyte subsets in our age groups.

### Target and pathway analysis of age-associated miRNAs

Each miRNA can regulate numerous target genes and therefore has the potential to modulate multiple pathways. To explore what targets and pathways may be regulated by these age-associated miRNAs, we used the Target Scan Human 5.1 database to predict targets for each miRNA that we identified in this study. Surprisingly, Target Scan did not predict any targets for miR-1538 nor are there any published reports on this miRNA. Therefore, we excluded this miRNA from any further examination. Two members of the miR-103 gene family, miR-103 and miR-107, are both downregulated with age. miR-103 and miR-107 only differ by one nucleotide and are predicted to target the same genes. Thus, these miRNAs were combined in our subsequent analysis.

The predicted targets for each age-associated miRNA from Target Scan were used for Ingenuity Pathway Analysis (IPA) to reveal the diseases, molecular functions, physiological systems and canonical pathways associated with each miRNA (Table 2). This analysis identified cancer as the most common disease among these miRNAs, which is not unexpected, as the most potent risk factor for cancer is advancing age. Cardiovascular disease was also another prominent disease. The targets for the age-associated miRNAs were most prominently predicted to function in gene expression. However, very little overlap was observed between the molecular functions and pathways for these miRNAs, suggesting that these miRNAs may regulate multiple pathways that influence diseases, such as cancer.

**Table 2:** Ingenuity analysis of microRNAs significantly downregulated with age.

	miR-103/107	miR-128	miR-130a	miR-155
**Diseases and Disorders**	Cardiovascular disease	Genetic disorder	Cancer	Hematological Disease
	Genetic Disorder	Neurological Disease	Cardiovascular disease	Cancer
**Molecular and**	Gene Expression	Gene expression	Cell death	Gene Expression
**Cellular Functions**	Cellular Movement	Amino Acid Metabolism	Cellular Movement	Cellular Development
**Physiological Systems**	Nervous System Development and Function	Organismal Development	Organismal Development	Organismal Development
	Behavior	Organ Development	Organ Development	Hematological System Development and Function
**Canonical Pathways**	Ran signaling	Molecular Mechanisms of Cancer	Pantothenate and CoA Biosynthesis	T cell Receptor Signaling
	Wnt/β-catenin Signaling	PPARa/RXR Activation	Human Stem Cell Pluripotency	Prolactin Signaling

	miR-24	miR-221	miR-496	
**Diseases and Disorders**	Cancer	Connective Tissue Disorder	Organismal Injury and Abnormalities	
	Neurological Disease	Cancer	Respiratory Disease	
**Molecular and**	Amino Acid Metabolism	Cellular Development	Carbohydrate Metabolism	
**Cellular Functions**	Post-translational Modification	Cell Cycle	Small Molecule Biochemistry	
**Physiological Systems**	Cardiovascular System Development and Function	Lymphoid Tissue Structure and Development	Cell-mediated Immune Response	
	Connective Tissue Development and Function	Nervous System Development and Function	Hematological System Development and Function	
**Canonical Pathways**	PTEN Signaling	Clathrin-mediated Endocytosis	Growth Hormone Signaling	
	Axonal Guidance Signaling	ERK/MAPK Signaling	Wnt/β-catenin Signaling	

The predicted targets (from Targetscan) were compiled for each respective miRNA and were examined using Ingenuity software. The top two pathways for each parameter are listed.

**Figure 4:**
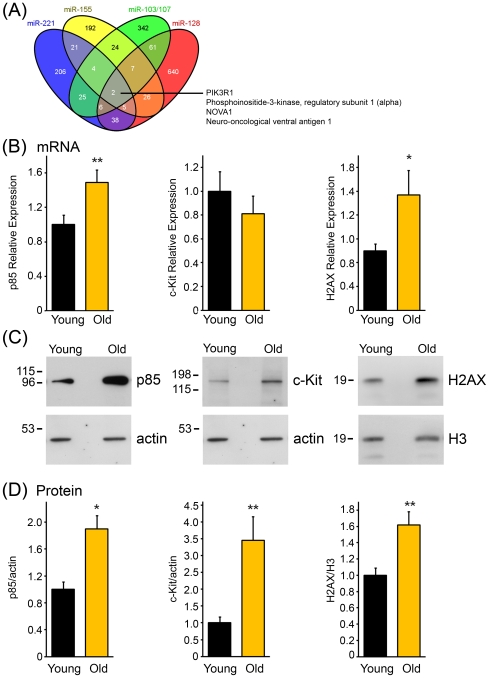
Putative target validation of miRNAs downregulated by age. (A) A Venn diagram showing the overlap between predicted targets of cancer-related miRNAs that were found to be downregulated in older participants. All cancer-related miRNAs have in common the predicted targets PIK3R1 and NOVA1. (B) Relative mRNA expression of PI3K, c-Kit and H2AX from 14 young and 14 old participants using real-time RT-PCR. The histograms show the normalized means ± SEM. (C) Representative immunoblots showing protein expression of PI3K, c-Kit and H2AX in a young and old patient. Participant PBMCs were lysed and immunoblotted with anti-PI3K, anti-c-Kit and anti-H2AX antibodies and reprobed with anti-actin and anti-histone H3 antibodies as protein loading controls. The same individuals were used for each representative immunoblot in C. (D) The protein levels of PI3K, c-Kit and H2AX from PBMCs were quantified from immunoblots and normalized to the amount of actin or histone H3. The histogram represents the normalized relative mean ± SEM from 14 young and 14 old individuals for PI3K and 10 young and 10 old individuals for c-Kit and H2AX. **P<0.01, *P<0.05 by Student's t-test.

Given the fact that IPA revealed that cancer was the most prominent disease linking these age-associated miRNAs and that 5 out of the 9 age-associated miRNAs have been previously reported to play a role in various cancers (Fig. 3C), we initially focused on these age-associated miRNAs with oncogenic (oncomirs) or tumor suppressive (ts-mirs) functions. To gain a better understanding of what targets may be regulated by these cancer-related miRNAs, we compared the overlap between the predicted targets for these miRNAs. Consistent with the variability in overlapping molecular pathways in our IPA, there was little overlap between the predicted targets among the age-associated oncomirs/ts-mirs (Fig. 4A). However, two predicted targets were in common between the miRNAs, PIK3R1 Phosphoinositide-3 kinase, regulatory subunit 1 alpha, and NOVA1 Neuro-oncological ventral antigen 1. NOVA1 is a brain-specific protein. We chose to focus our further investigations on PIK3R1 which encodes the regulatory p85α subunit of phosphoinositide 3-kinse (PI3K), a lipid kinase that plays a key role in many cellular processes including cell survival, cell proliferation, and tumorigenesis [24], [25].

### Upregulation of PI3 Kinase expression with age

We examined whether the expression of PI3K may be altered in young and old participants, consistent with the expression of the age-associated miRNAs predicted to target this protein. Because in most cases miRNAs downregulate the expression of their target genes by inducing cleavage and degradation of mRNAs or by inhibiting protein translation [4], [5], and we observed downregulation of these cancer-related miRNAs in older individuals, we hypothesized that the levels of PI3K would be elevated in older participants. Indeed, we found by real-time RT-PCR that mRNA levels of the p85α subunit of PI3K were significantly higher in older participants than younger participants (Fig. 4B). Immunoblotting experiments confirmed that p85α protein levels were elevated in older individuals (Fig. 4B, 4C), suggesting that age-associated miRNAs may affect p85α by modulating both p85α mRNA stability and translation. In addition to the p85α regulatory subunit, PIK3R1 also encodes its splice variants p55α and p50α, which lack the N-terminal SH3 and BCR homology domains [24]. Using primers specific for these isoforms, we did not observe any significant changes in expression of p55α or p50α mRNA in old participants (data not shown). In addition, p55α and p50α proteins were expressed at low levels in PBMCs from these individuals and were not significantly altered among the different age groups (data not shown). Therefore, the age-dependent upregulation of the regulatory subunit of Class 1A PI3K appears to be specific to the p85α subunit and this expression correlates with downregulation of the age-associated miRNAs.

**Figure 5:**
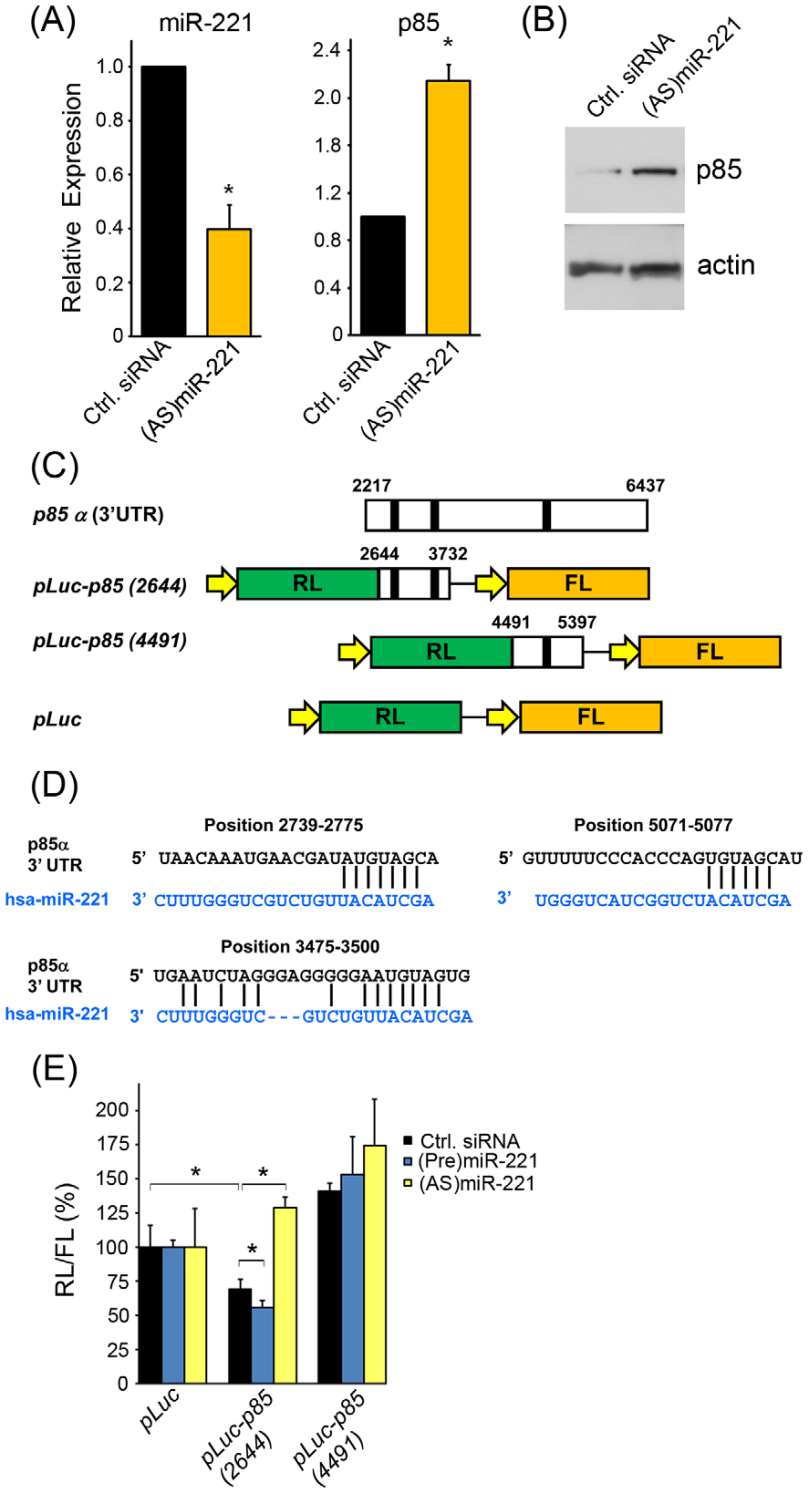
miR-221 regulates p85 expression. (A) Hela cells were transfected with control (Ctrl) siRNA or (AS)miR-221 and 48 h later mRNA levels of miR-221 and p85 were analyzed by RT-qPCR using U6 and UBC for normalization, respectively. The histogram represents the mean ± SEM from three independent experiments. *P<0.05 by Student's t-test. (B) Forty eight hrs after transfection with the indicated small RNAs, protein lysates were used for Western blot analysis of p85α expression. β-actin signals served as protein loading controls. (C) Schematics of the *p85α* 3′UTR and the dual-luciferase plasmids used in (E). *pLuc* control plasmid expresses both the *Renilla* luciferase (RL) and firefly luciferase (FL). Constructs of the *p85α* 3′ UTR span either two (*pLuc-p85 (2644)*) or one (*pLuc-p85 (4491)*) of the predicted miR-221 sites (black bars). (D) The *p85α* mRNA was predicted to be targeted by miR-221 using prediction algorithims from Target Scan and microRNA.org. The position of each predicted site within *p85α* is indicated. (E) Hela cells were transfected with the dual-luciferase plasmids shown and either control siRNA, (Pre)miR-221 or (AS)miR-221. The ratio of the RL/FL activity is shown. *P<0.05 using one-way ANOVA and Tukey's post-hoc test for the indicated comparisons.

To determine whether the age-associated miRNAs regulate PI3K expression, we focused on miR-221 since this miRNA had the highest prediction score in Target Scan to target p85α. We lowered the levels of miR-221 using an *antagomir*, an RNA complementary (antisense, AS) to miR-221, to mimic the change in expression of miR-221 with age. Transfection with (AS)miR-221 reduced miR-221 expression, and increased both the mRNA and protein levels of p85α (Fig. 5A,B). This suggests that miR-221 regulates the expression of p85α and that this miRNA may modulate p85α levels during the aging process.

In order to determine whether miR-221 regulates the *p85α* 3′UTR, we generated dual-luciferase constructs (Fig. 5C). Computational algorithms predicted three different miR-221 sites in the *p85α* 3′ UTR (Fig. 5C,D). Therefore, we made two different constructs that span the predicted miR-221 target sites (*pLuc-p85 (2644)* and *pLuc-p85 (4491)*) and cloned them 5′ to the *Renilla* luciferase (RL) reporter. The plasmid contains a second reporter, firefly luciferase (FL), which is driven from a separate promoter and serves as a transfection control. We measured the ratios of RL/FL in Hela cells transfected with the different dual-luciferase constructs and either control siRNA, precursor (Pre)miR-221, or (AS)miR-221. Transfection of the *pLuc-p85 (2644)* plasmid reduced the activity of the luciferase reporter compared to pLuc control, indicating that either or both of the first two predicted sites in the *p85α* 3′ UTR are targeted by endogenous miRNAs to repress the translation of *p85α* mRNA (Fig. 5E). Elevating the levels of miR-221 using (Pre)miR-221 further reduced luciferase activity compared to control siRNA, while reducing the levels of miR-221 by transfection of (AS)miR-221 increased the expression of the reporter above control (Fig. 5E). These findings suggest that the effects observed on RL/FL activity for the *pLuc-85 (2644)* plasmid depend on the presence of miR-221. Interestingly, RL/FL ratios are not affected when cells are transfected with the *pLuc-p85 (4491)* plasmid with or without the different miRNAs, indicating that this predicted miR-221 site is not important for miRNA-mediated translation repression of *p85α* mRNA (Fig. 5E).

### Targets of age-associated miRNAs are differentially expressed with age

To examine if expression of other target genes may be altered in older participants, we focused on previously reported targets for miR-24 and miR-221, two age-associated miRNAs we identified in this study. It has been demonstrated that miR-221 regulates expression of the oncogene c-Kit [26], a receptor tyrosine kinase that is overexpressed and/or highly active in various leukemias and other cancers [27], [28]. We found that c-Kit protein levels were higher in older individuals (Fig. 4B–D), but that c-Kit mRNA levels were not changed between the different groups, consistent with the fact that miR-221 has been reported to predominantly regulate c-Kit translation rather than c-Kit mRNA levels [26]. miR-24 has been reported to modulate the expression of histone H2AX [29], which is a key component in the repair of DNA double-strand breaks. It has been reported that phosphorylation of H2AX (referred to as γ-H2AX) increases with age in human donor PBMCs [30]. We found that H2AX expression was significantly higher at both the mRNA and protein levels in older participants (Figure 4B-D). This suggests that in addition to the occurrence of γ-H2AX, expression of H2AX may also be a molecular indicator of age.

## Discussion

Despite the accumulating evidence linking miRNAs to various age-related diseases, very little is known about how these small RNAs contribute to the aging process. Here, we have surveyed expression of miRNAs from young and old individuals in order to provide a genome-wide assessment of miRNA expression during human aging. We chose to use a miRNome real-time RT-PCR based approach instead of microarray for several reasons. In general, real-time RT-PCR is more quantitative, accurate and often microarray data need validation by RT-PCR. We used this initial profiling to identify miRNAs that exhibit age-associated changes in expression in two different sets of young and old individuals and further validated the abundance of these miRNAs in a larger cohort. We identified 9 miRNAs that were significantly downregulated with age in a larger number of individuals, suggesting that miRNAs may be important regulators of lifespan.

Interestingly, we found in our screen of over 800 human miRNAs that the majority of miRNAs are downregulated with age (Fig. 1,2). In comparing the two different miRNome analyses (which represent different young and old participants) 16.5% of the miRNAs decline in abundance, whereas 2.5% increase in abundance 2-fold (Fig. 2). These findings are comparable to prior microarray studies where the majority of miRNAs decline in abundance over the lifespan of *C. elegans* [10]. Given this similarity and the fact that many of the human miRNAs are evolutionarily conserved, miRNAs may serve as key regulators of age-related decline across species. Nonetheless, it is important to study miRNAs in the context of human aging. In agreement with the increased complexity of higher organisms, currently there are approximately 6X more miRNAs in humans than in worms (∼851 versus ∼136)(MicroCosm Targets).

In addition to *C. elegans*, changes in miRNA expression have been observed with aging in the mouse. Specifically, in the mouse brain 31 miRNAs are significantly upregulated and 17 are downregulated with age. The majority of the upregulated miRNAs are predicted to target and correlate in expression with components of the mitochondrial complexes [13]. In the mouse liver, several miRNAs found to be upregulated were associated with targeting mRNAs important for detoxification and regeneration of the liver [11]. These findings point to the fact that upregulation of specific miRNAs may target particular genes that are important for tissue-specific aging decline. Interestingly, miR-27a was found to be significantly upregulated in the Ames dwarf mouse model of aging, which have extended lifespan [12]. Reduced expression of mir-27a was observed in control older mice and was also found to be decreased in older individuals in our miRNome analyses, suggesting that perhaps this miRNA may be important for regulating longevity in both mice and humans and may be an interesting miRNA to pursue in future studies.

It is interesting to note that a decrease in miRNA expression is also observed during cellular senescence in human tissue culture cells grown *in vitro*. Senescent cells accumulate with advancing age in humans, primates and rodents, and are often found in tissues of age-associated pathologies [15]. Furthermore, the data suggest that senescent cells play a role in tumor suppression as well as a potential, albeit controversial, role in contributing to the aging process [15], [31]. One characteristic of replicative senescence is a decrease in protein translation. It is tempting to speculate that decreased levels of miRNAs in older individuals, similar to senescent cells, may help cells to maintain elevated protein levels at a time when translation is depressed due to advancing age, perhaps as an energy-saving or compensatory mechanism.

Several senescence-associated miRNAs and their targets have been identified. In our studies, we found that several miRNAs have similar expression patterns in both senescent cells and in aging PBMCs, including miR-17, miR-24, miR-93, miR-141, miR-146, miR-155 and miR-106a. However, several senescence-associated miRNAs (miR-15b, miR-25, miR-106b) are either not changed significantly or showed more interindividual variability in our miRNome analysis, suggesting that some but not all miRNAs expression patterns are consistent between senescent cells and aged cells. This discrepancy could also be reflected in the different cell types examined as it is known that some miRNAs show tissue specificity. Based on our miRNome data, we further verified the expression of miR-24 and miR-155 and showed that these miRNAs are downregulated with age in our larger cohort (Fig. 3). Intriguingly, it has been shown that miR-24 can target E2F2, p16^INK4a^, MKK4 and H2AX [17], [19], [29], [32]. We found that levels of H2AX mRNA and protein are higher in older individuals. This finding is interesting because most studies have focused on measuring the levels of γ-H2AX, a posttranslational modification of H2AX that marks DNA double-strand breaks [33]. It has been reported that γ-H2AX staining increases with age in human PBMCs, in mouse tissues and with cellular senescence [30], [34]. It is interesting to propose that with increasing age miR-24 coordinately modulates H2AX expression, in part to help to overcome the additional oxidative stress and DNA damage that occurs with age. Further studies will be needed to validate if increased expression of H2AX is indeed a biomarker of age, in addition to, γ-H2AX.

Although we found that miR-155 decreased with age and others have shown it decreased during cellular senescence [16], miR-155 is widely accepted as an oncogenic miRNA that has been found to be upregulated in various cancers [4], [35]. Because miR-155 is localized to a fragile locus (termed the B cell integration cluster *BIC*) that is an integration site of viral-induced lymphomas, it is possible that expression of miR-155 is regulated differently from other miRNAs. Consistent with this idea, expression of the *BIC* itself does not always correlate with expression of miR-155 in lymphoma cell lines [36], further suggesting that we do not fully understand how levels of miR-155 are regulated.

In addition to the miR-155 oncomir, we found that several other cancer-associated miRNAs are downregulated with age including miR-103, miR-107, miR-128 and miR-221 (see Fig. 3C for list of cancers associated with each miRNA). Although specific miRNAs have been reported to be upregulated in cancer, most miRNAs are generally downregulated in human tumors [37], [38]. Global inhibition of miRNAs enhances both cellular transformation and tumorigenesis [39], further supporting a tumor-promoting role for miRNA loss of function. In agreement of this idea, expression of individual miRNAs repressed Myc-induced tumorigenesis and viral introduction of let-7 suppressed tumor growth in a mouse model of lung adenocarcinoma [40], [41], [42]. These studies underscore the importance of miRNA expression in age-related diseases, such as cancer, and open the possibility that miRNAs can be used for cancer therapeutics.

To determine if miRNAs would have therapeutic potential, it is important to identify the targets for each miRNA. This is a challenging task, since a single miRNA can regulate numerous targets and a single gene can be targeted by multiple miRNAs. To this end, we used Target Scan and Ingenuity Pathway Analysis to decipher the predicted targets and pathways for the age-associated oncomirs/ts-mirs identified in our study. Remarkably, only 2 targets overlapped between these miRNAs, NOVA1 and PIK3R1. We focused on PIK3R1, which encodes the p85α subunit of PI3K, a well-established integrator of multiple signaling pathways that promote tumorigenesis. We found that suppression of the age-associated miR-221 increases the abundance of p85 mRNA and protein (Fig. 5). As changes in miR-221 expression have been described in various cancers [4], [35], [43], and in the context of age in this report, miR-221 may be a modulator of pathways important for aging and tumorigenesis.

Interestingly, not only is PI3K important for human cancer but is also part of the IGF-1 signaling pathway, which plays a well-established role in longevity [2]. Moreover, mutations in the *age-1* gene, which encodes a class 1 PI3K catalytic subunit, extends the lifespan of *C. elegans* [44]. One caveat of the IGF-1 pathway is that reduced signaling increases longevity, whereas, enhanced signaling promotes cancer. It is possible that the aberrant expression of different subunits of PI3K, as we have observed in this study, may tip the balance to enhance or inhibit cancer progression during the aging process. In support of this idea, upregulation of the PI3K p110 subunit was recently reported in cancer stem cells and activation of this pathway enhanced the tumorigenic potential of these cells [45]. It would be interesting to examine if increased levels of the PI3K p85 subunit are also observed in cancer stem cells.

In addition to PI3K, we also examined expression of the miR-221 target c-Kit in young and old individuals. Higher levels of c-Kit protein were observed in older individuals, whereas, c-Kit mRNA levels were unchanged between the age groups. These data are consistent with a previous report that miR-221 primarily regulates translation of c-Kit rather than c-Kit mRNA levels [26]. c-Kit is overexpressed and/or activated in various leukemias and other cancers [27], [28]. For example, it has been predicted that c-Kit is expressed in 80–90% of acute myelogenous leukemia (AML) cases and that coordinated activation of the c-Kit pathway and other oncogenic signaling pathways contribute to the severity of this cancer and other types of leukemias [27], [28]. One interesting observation is that PI3K is downstream of the c-Kit receptor tyrosine kinase. Therefore, it is interesting to hypothesize that upregulation of PI3K may help to potentiate c-Kit signaling in hematopoietic cells with advancing age, and perhaps, this enhanced signaling primes the cells for oncogenic transformation [46].

Several of the age-associated miRNAs that we identified have not been well-characterized including miR-130a, miR-496, and miR-1538. Specifically, there are currently no published reports or targets predicted yet in TargetScan or Pictar for miR-1538. In the future, it will be interesting to examine how these underexplored miRNAs contribute to the aging process.

Our cohort contains subjects of different races and sexes, suggesting that downregulation of age-associated miRNAs may occur regardless of both sex and race. Consistent with this idea, three-way analysis of variance analysis using sex, race and age as variables revealed that only miR-299-3p was significantly altered in different sexes, expression is lower in males than females (data not shown). Localized on chromosome 14q101, miR-299-3p was recently proposed to be part of a miRNA signature that predicts estrogen receptor expression in breast cancer patients, indicating that this miRNA may play a role in hormonal regulation in normal tissues and during oncogenesis [47].

In summary, we have identified several miRNAs that are downregulated with human age. The loss of miRNA function during the aging process may be due to transcriptional repression, deletion, mutation, epigenetic silencing, or aberrant miRNA processing [4], [48]. Future work will determine how these age-associated miRNAs decrease in abundance with age. Nevertheless, it is important to determine the expression of miRNAs and their targets with human age, as we have done here, and to follow-up with a systematic analysis of the pathways regulated by each miRNA in aging cells. A critical understanding of the precise role each miRNA plays in the aging process will hopefully aid in the development of new anti-aging therapies and in determining if miRNAs and their targets could be used as diagnostic or prognostic indicators of age or age-related diseases.

## Materials and Methods

### Study Participants

Fasting blood samples were obtained from participants in the Healthy Aging in Neighborhoods of Diversity across the Life Span (HANDLS) study of the National Institute on Aging Intramural Research Program (NIA IRP). The purpose of this study is to unravel the effects of race and socioeconomic status on the development of age-associated health disparities. The cohort consists of Whites and African Americans between the ages of 30–64 residing in Baltimore, Maryland. The study has been approved by the Institutional Review Board of the Med-Star Research Institute and all participants provided written informed consent. The demographics of the study sub-population presented in Table 1 were collected from interviews and self-reported medical history. For this cohort, we excluded participants with documented Hepatitis B, Hepatitis C or human immunodeficiency virus (HIV) infection.

### Peripheral blood mononuclear cells (PBMCs) isolation

Blood samples collected in 8-ml Vacutainer® heparinized vials (BD, Franklin Lakes, NJ) were transported to the laboratory at room temperature and peripheral blood mononuclear cell (PBMC) isolation was performed within 3 hours of phlebotomy. Blood was diluted with RPMI-1640 medium and Histopaque 1077 (Sigma-Aldrich, St. Louis, MO) was added to the bottom of the tubes. Samples were centrifuged at 200 x *g* for 15 min and the interphase layer containing PBMCs were collected and furthered washed with RPMI-1640 medium. The cell pellets were resuspended in freezing medium containing 40% RPMI-1640, 50% FBS and 10% DMSO, aliquoted and stored at −80°C.

### Quantitative miRNome miRNA profiling

Cryopreserved PBMCs were thawed quickly in the presence of 0.2 ml PBS and RNaseOUT™ (Invitrogen Carlsbad, CA) and centrifuged at 1000 x *g* for three minutes. Total RNA was isolated using TRIzol® (Invitrogen, Carlsbad, CA) and DNase treated (Applied Biosystems, Foster City, CA) according to manufacturer's directions. RNA was quantified and assessed using both a NanoDrop ND-1000 Spectrophotometer and the RNA 6000 Nano Total RNA Assay and Agilent Techonologies 2100 Bioanalyzer. We used the miRNome miRNA profiling kit from System Biosciences (Mountain View, CA) to examine miRNA expression in two young and two old participants (see Table 1 for demographics) in two different experiments. Four micrograms of RNA was reverse transcribed using QuantiMir™ cDNA techonology, which tags and converts small RNAs into cDNAs. miRNA profiling was performed according to manufacturer's directions with some exceptions. In brief, 5 µl of Mastermix (all the synthesized cDNA, SYBR Green master mix, Universal Reverse Primer and nuclease free water) was added to a 96-well plate containing 3 µl of the manufacturer's human miRNA assay primers (diluted 1∶5). cDNA for one young and one old individual were run on each plate on an Applied Biosystems 7500 Real-Time PCR machine according to standard procedures. Samples were normalized to U1 expression and analyzed using the ΔΔCT method. Similar results were observed with other normalization genes.

### Real-time RT-PCR

Total RNA from 14 young and 14 old subjects (see Table 1 for demographics) were isolated and analyzed as above. cDNA was synthesized using the QuantiMir™ cDNA Kit (System Biosciences, Mountain View, CA). Forward primers were designed to be the exact sequences of the miRNAs listed in the miRBase database (http://www.mirbase.org) and are listed in Table S4. An Universal Reverse Primer was supplied by the manufacturer. For gene expression analysis, gene-specific primer pairs for H2AX were described previously [29] and other primers are as follows: PI3K p85α subunit isoform 1 forward 5′-AGCAACCTGGCAGAATTACGA and reverse 5′- AAACGTGCACATCGATCATTTC and for c-Kit 5′-TTGTGATTTTGGTCTAGCCAGAGA and reverse 5′-GTGCCATCCACTTCACAGGTAG. Real-time RT-PCR reactions were performed according to the manufacturer's (System Biosciences, Mountain View, CA) directions on an Applied Biosystems 7500 Real-Time PCR System, with the exception that 6 times more cDNA was needed to detect c-Kit expression. miRNAs were normalized to the average of three different miRNAs miR-147, miR-574-3p and miR-1469. The expression of these miRNAs did not change in the two miRNome analysis and also showed minimal variability in expression among all the participants. Similar results were also obtained when we normalized to the snoRNAs RNU24, RNU49 and U47. H2AX, c-Kit and PI3K were normalized to the average of HPRT and UBC expression, which were two reference genes that were found to be the least variable reference genes in the participant PBMCs.

The TaqMan® microRNA assay (Applied Biosystems) was used to quantitate miR-24 and miR-221 expression in young and old participants and normalized to miR-147 expression. For duplicate wells, a total of 100 ng of RNA was used for miR-24 and miR-147 and 200 ng of RNA for miR-221.

### Bioinformatics

Each age-associated miRNA was tested using Target Scan, which uses a computational algorithm to identify predicted mRNA targets. All the predicted targets from this analysis were then used as input for Ingenuity Pathway Analysis. IPA scores the list of genes for each miRNA against the canonical pathways in the Ingenuity Knowledge Base Genes Only reference set and default settings were used throughout the analysis. IPA has key components for Signaling and Metabolic pathways analysis, Cellular and Disease Process Analysis, Molecular Network Analysis and Contextual Data analysis. For each analysis, IPA ranks each function or pathway using the right-tailed Fisher Exact Test, which measures the likelihood that the lists of genes are associated with a certain pathway/function (www.ingenuity.com). For each miRNA, the top rank for each analysis was used for Table 2.

### Immunoblotting of Protein Extracts from Patients

Cryopreserved PBMCs were thawed quickly, washed with cold PBS and spun at 1000 x *g* for three minutes. Cell pellets were resuspended in 2X Laemmli sample buffer, separated by SDS-PAGE and probed by immunoblotting with anti-Histone H2AX polyclonal antibodies (Millipore, Billerica, MA), p85α PI3K monoclonal antibodies (BD Biosciences, San Jose, CA), and c-Kit goat polyclonal antibodies (R&D systems, Minneapolis, MN). Membranes were stripped and reprobed with anti-actin goat polyclonal antibodies (Santa Cruz Biotechnology, Santa Cruz, CA) and anti-Histone H3 antibodies (Millipore, Billerica, MA) as protein loading controls. Immunoblots were quantified using ImageJ.

### Cell culture, transfection, and small RNAs

HeLa cells were cultured in DMEM containing 10% FBS and transfected using Lipofectamine 2000 (Invitrogen, Carlsbad, CA). Anti-miR™ miRNA inhibitor against miR-221 ((AS)miR-221) or FAM™-labeled Anti-miR negative control (Ctrl siRNA) were used at a final concentration of 100 nM (Ambion, Austin, TX). RNA and protein were isolated from the cells forty eight hours after transfection as described above.

### Luciferase Assays

Plasmids for reporter analysis were generated from the dual-luciferase reporter vector psiCHECK™-2 from Promega (referred to as *pLuc*). The *p85α* 3′ UTR luciferase plasmids containing predicted miR-221 sites (Fig. 5C) were generated by PCR using the following primers: p85 (2644) F: CTCGAGgtttggtccagcctggttta and R: GCGGCCGCcaaacttggcacttccttcc and p85 (4491) F: CTCGAGctctcatcgccagacaactg and R: GCGGCCGCgcaacctcaaagggaaaaatc (uppercase letters, Xho1 and Not1 sites). Hela cells were transfected with 100 ng of the luciferase plasmids and transfected 24 hrs later with 100 nM Ctrl siRNA, (AS)miR-221 or (Pre)miR-221 (Ambion, Austin, TX). After 24 hrs, the ratio of RL and FL activities were measured using the dual-luciferase kit (Promega, Madison, WI) according to the manufacturer's instructions.

## Supporting Information

Table S1 miRNome analysis of miRNA expression in young versus old participants.
(0.11 MB XLS)

Table S2 Fold change of the top upregulated and top downregulated miRNAs in the miRNome analysis.
(0.04 MB XLS)

Table S3 miRNome expression values for microRNAs examined in Fig. 3 and Fig. S1.
(0.02 MB XLS)

Table S4 Forward primer sequences for validated microRNAs.
(0.05 MB DOC)

Figure S1 Real-time RT-PCR results for changes in miRNA expression in young and old participants. Expression of miRNAs from the miRNome analysis were further validated in 14 young and 14 old patient PBMCs (see Table 1B for detailed demographic data) using real-time RT-PCR as described in Materials and Methods. The histograms show normalized averages + SEM from duplicate experiments. ##P = 0.05 by Student's t-test and P = 0.04 by three-way ANOVA, # P = 0.08 by Student's t-test.
(3.73 MB TIF)

Figure S2 Lymphocyte marker expression in participant PBMCs. CD4, CD8 and IL-7 receptor expression was examined in young and old individuals using RT-qPCR and normalized to the average of HPRT and UBC. The ratio of CD4/CD8 expression is also shown. The indicated P values show the significance of each parameter between young and old individuals using Student's t-test.
(1.23 MB TIF)
