# Supplementary material for: microRNA Expression Patterns Reveal Differential Expression of Target Genes with Age
Source: PLoS One. 2010 May 20;5(5):e10724. doi: 10.1371/journal.pone.0010724 (PMC2873959; doi:10.1371/journal.pone.0010724)
Supplement: Table S4 — Forward primer sequences for validated microRNAs. (0.05 MB DOC) [file pone.0010724.s004.doc]

| **Supplementary Table 4.** Forward primer sequences for validated microRNAs. | | | |
| --- | --- | --- | --- |
| **miRBase Accn #** | **mature miRNA ID** | **DNA sequence** | **# bases** |
| MIMAT0000254 | hsa-miR-10b | TACCCTGTAGAACCGAATTTGTG | 23 |
| MIMAT0000104 | hsa-miR-107 | AGCAGCATTGTACAGGGCTATCA | 23 |
| MIMAT0000101 | hsa-miR-103 | AGCAGCATTGTACAGGGCTATGA | 23 |
| MIMAT0000424 | hsa-miR-128 | TCACAGTGAACCGGTCTCTTT | 21 |
| MIMAT0000646 | hsa-miR-155 | TTAATGCTAATCGTGATAGGGGT | 23 |
| MIMAT0000425 | hsa-miR-130a | CAGTGCAATGTTAAAAGGGCAT | 22 |
| MIMAT0000080 | hsa-miR-24 | TGGCTCAGTTCAGCAGGAACAG | 22 |
| MIMAT0000278 | hsa-miR-221 | AGCTACATTGTCTGCTGGGTTTC | 23 |
| MIMAT0002818 | hsa-miR-496 | TGAGTATTACATGGCCAATCTC | 22 |
| MIMAT0007400 | hsa-miR-1538 | CGGCCCGGGCTGCTGCTGTTCCT | 23 |
| MIMAT0000063 | hsa-let-7b | TGAGGTAGTAGGTTGTGTGGTT | 22 |
| MIMAT0004483 | hsa-let-7c* | TAGAGTTACACCCTGGGAGTTA | 22 |
| MIMAT0004486 | hsa-let-7f-1* | CTATACAATCTATTGCCTTCCC | 22 |
| MIMAT0004489 | hsa-miR-16-1* | CCAGTATTAACTGTGCTGCTGA | 22 |
| MIMAT0004674 | hsa-miR-30c-1* | CTGGGAGAGGGTTGTTTACTCC | 22 |
| MIMAT0004506 | hsa-miR-33a* | CAATGTTTCCACAGTGCATCAC | 22 |
| MIMAT0004517 | hsa-miR-106a* | CTGCAATGTAAGCACTTCTTAC | 22 |
| MIMAT0000443 | hsa-miR-125a-5p | TCCCTGAGACCCTTTAACCTGTGA | 24 |
| MIMAT0000770 | hsa-miR-133b | TTTGGTCCCCTTCAACCAGCTA | 22 |
| MIMAT0000257 | hsa-miR-181b | AACATTCATTGCTGTCGGTGGGT | 23 |
| MIMAT0004567 | hsa-miR-219-1-3p | AGAGTTGAGTCTGGACGTCCCG | 22 |
| MIMAT0000276 | hsa-miR-219-5p | TGATTGTCCAAACGCAATTCT | 21 |
| MIMAT0004679 | hsa-miR-296-3p | GAGGGTTGGGTGGAGGCTCTCC | 22 |
| MIMAT0000687 | hsa-miR-299-3p | TATGTGGGATGGTAAACCGCTT | 22 |
| MIMAT0000762 | hsa-miR-324-3p | ACTGCCCCAGGTGCTGCTGG | 20 |
| MIMAT0004700 | hsa-miR-331-5p | CTAGGTATGGTCCCAGGGATCC | 22 |
| MIMAT0000754 | hsa-miR-337-3p | CTCCTATATGATGCCTTTCTTC | 22 |
| MIMAT0001541 | hsa-miR-449a | TGGCAGTGTATTGTTAGCTGGT | 22 |
| MIMAT0004772 | hsa-miR-499-3p | AACATCACAGCAAGTCTGTGCT | 22 |
| MIMAT0006778 | hsa-miR-516a-3p | TGCTTCCTTTCAGAGGGT | 18 |
| MIMAT0003255 | hsa-miR-588 | TTGGCCACAATGGGTTAGAAC | 21 |
| MIMAT0003264 | hsa-miR-596 | AAGCCTGCCCGGCTCCTCGGG | 21 |
| MIMAT0003271 | hsa-miR-603 | CACACACTGCAATTACTTTTGC | 22 |
