# Supplementary figures and images for: microRNA Expression Patterns Reveal Differential Expression of Target Genes with Age
Source: PLoS One. 2010 May 20;5(5):e10724. doi: 10.1371/journal.pone.0010724 (PMC2873959; doi:10.1371/journal.pone.0010724)

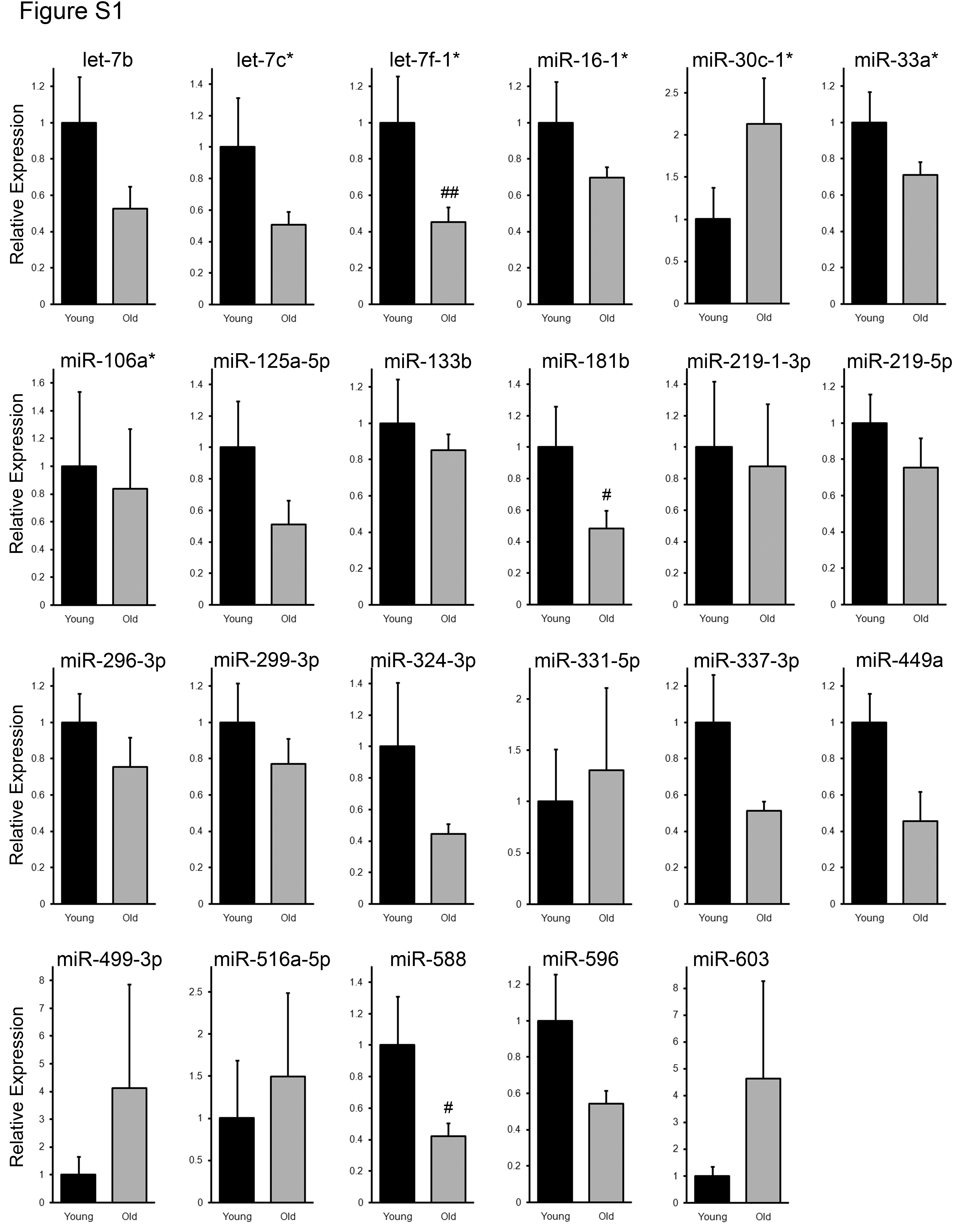

Supplement: Figure S1 — Real-time RT-PCR results for changes in miRNA expression in young and old participants. Expression of miRNAs from the miRNome analysis were further validated in 14 young and 14 old patient PBMCs (see Table 1B for detailed demographic data) using real-time RT-PCR as described in Materials and Methods. The histograms show normalized averages + SEM from duplicate experiments. ##P = 0.05 by Student's t-test and P = 0.04 by three-way ANOVA, # P = 0.08 by Student's t-test. (3.73 MB TIF) [file pone.0010724.s005.tif]

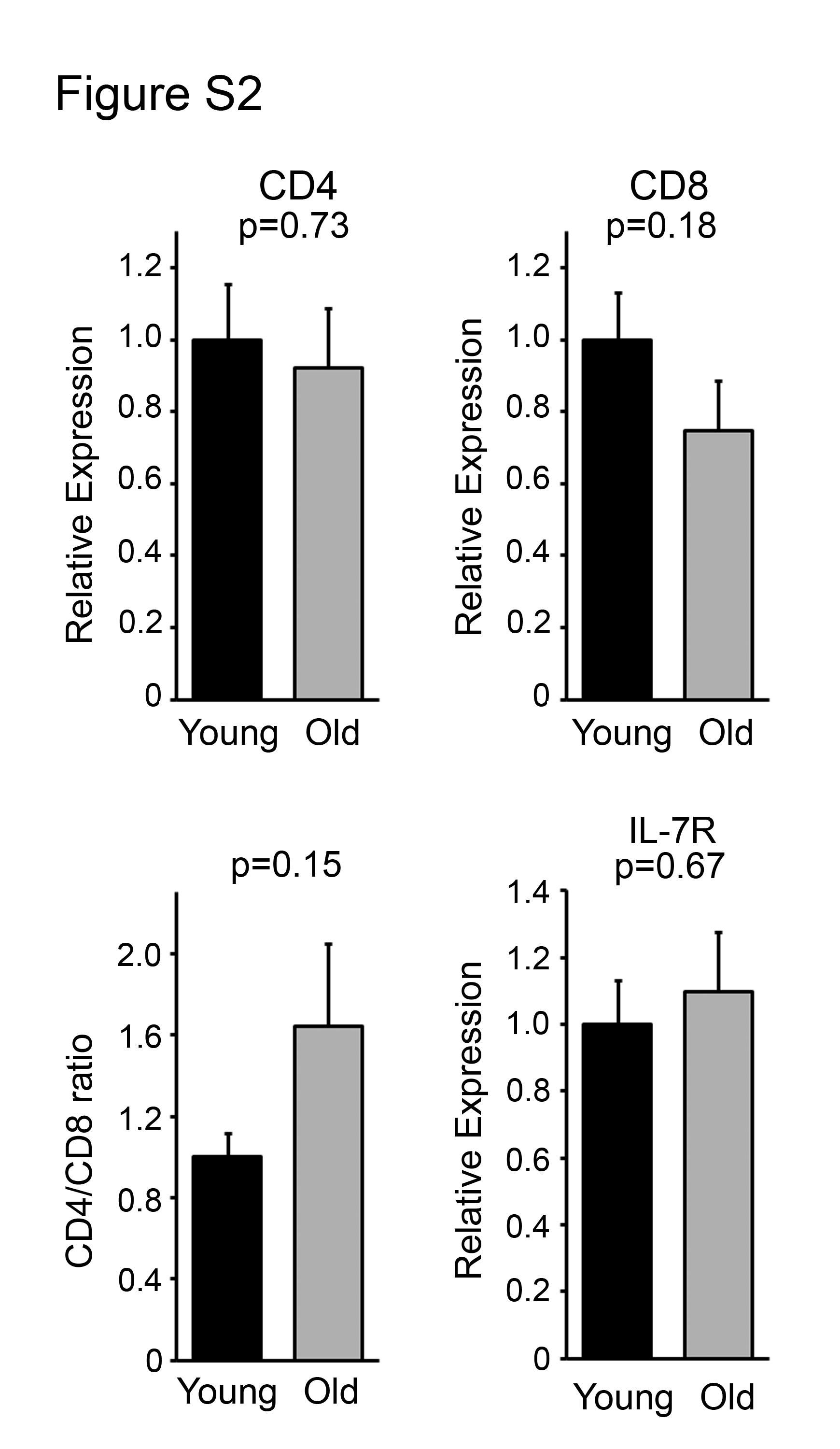

Supplement: Figure S2 — Lymphocyte marker expression in participant PBMCs. CD4, CD8 and IL-7 receptor expression was examined in young and old individuals using RT-qPCR and normalized to the average of HPRT and UBC. The ratio of CD4/CD8 expression is also shown. The indicated P values show the significance of each parameter between young and old individuals using Student's t-test. (1.23 MB TIF) [file pone.0010724.s006.tif]
